# GWAS Meta-Analysis Reveals Shared Genes and Biological Pathways between Major Depressive Disorder and Insomnia

**DOI:** 10.3390/genes12101506

**Published:** 2021-09-26

**Authors:** Yi-Sian Lin, Chia-Chun Wang, Cho-Yi Chen

**Affiliations:** 1 Institute of Biomedical Informatics, National Yang Ming Chiao Tung University, Taipei 11221, Taiwan; helen82323@gmail.com (Y.-S.L.); wangterry88@gmail.com (C.-C.W.); 2 Brain Research Center, National Yang Ming Chiao Tung University, Taipei 11221, Taiwan

**Keywords:** GWAS, MDD, insomnia, eQTL, comorbidity, STRING, gene network, meta-analysis

## Abstract

Major depressive disorder (MDD) is one of the most prevalent and disabling mental disorders worldwide. Among the symptoms of MDD, sleep disturbance such as insomnia is prominent, and the first reason patients may seek professional help. However, the underlying pathophysiology of this comorbidity is still elusive. Recently, genome-wide association studies (GWAS) have begun to unveil the genetic background of several psychiatric disorders, including MDD and insomnia. Identifying the shared genomic risk loci between comorbid psychiatric disorders could be a valuable strategy to understanding their comorbidity. This study seeks to identify the shared genes and biological pathways between MDD and insomnia based on their shared genetic variants. First, we performed a meta-analysis based on the GWAS summary statistics of MDD and insomnia obtained from Psychiatric Genomics Consortium and UK Biobank, respectively. Next, we associated shared genetic variants to genes using two gene mapping strategies: (a) positional mapping based on genomic proximity and (b) expression quantitative trait loci (eQTL) mapping based on gene expression linkage across multiple tissues. As a result, a total of 719 shared genes were identified. Over half (51%) of them are protein-coding genes. Functional enrichment analysis shows that the most enriched biological pathways are related to epigenetic modification, sensory perception, and immunologic signatures. We also identified druggable targets using a network approach. Together, these results may provide insights into understanding the genetic predisposition and underlying biological pathways of comorbid MDD and insomnia symptoms.

## 1. Introduction

Major depressive disorder (MDD) is one of the most prevalent and disabling mental disorders worldwide, with a lifetime prevalence of 15% [1]. Sleep disturbance is the core symptom of MDD that occurs in up to 90% of patients and is the first reason patients seek professional help [2,3]. Additionally, according to the DSM-5, the major depressive episode includes “insomnia or hypersomnia nearly every day” [4]. Insomnia is a sleep problem that involves individuals having difficulty sleeping and is often chronic, negatively affecting their quality of life [5]. On the other hand, studies have shown that people with insomnia are more likely to develop depression and increase suicidal ideation if diagnosed with MDD [6,7]. Moreover, drugs and behavioral treatments for comorbid MDD and insomnia symptoms can improve both outcomes [8,9]. Therefore, the relationship between MDD and insomnia may be bi-directional [10,11].

Although the underlying mechanism of MDD remains elusive, MDD is recognized as a complex disorder contributed by both genetic and environmental factors. The heritability of MDD is 40% to 50% suggested by twin studies [11]. However, the genetic component of insomnia is hard to estimate because it can coexist with other medical and psychiatric conditions. Recent genome-wide association studies (GWAS) have identified genetic variants for depression [12,13,14,15,16,17] and insomnia disorder [18,19,20,21,22,23], respectively. GWAS is a powerful approach to test genome-wide genetic variants of population-level to identify genotype–phenotype associations [24]. Notably, insomnia and MDD are genetically correlated as shown by Lane et al. (r_g_ = 0.34 and 0.24 in two studies) [18,21], Hammerschlag et al. (r_g_ = 0.41) [19], Stein et al. (r_g_ = 0.44) [20], and Jansen et al. (r_g_ = 0.59) [22,25]. Therefore, identifying the shared genomic risk loci between MDD and insomnia would be a valuable strategy to associate the underlying pathophysiology of MDD with insomnia.

In this study, we exploit this strategy to explore the shared candidate genes and related biological pathways involved in the pathogenesis of MDD and insomnia. First, we performed a GWAS meta-analysis of MDD and insomnia using the summary statistics from Wray et al. [16] and Lane et al. [21], respectively, to identify shared genetic links and new associated variants between the two psychiatric conditions. Next, to characterize the functional roles of the variants, we conducted positional and expression quantitative trait loci (eQTL) mapping followed by a series of functional enrichment analyses. Finally, to provide potential druggable targets of MDD with insomnia, we prioritized the genes (targets) based on their connectivity degree in the human protein–protein interaction (PPI) network and searched for potential drugs using the drug–gene interaction databases.

## 2. Materials and Methods

### 2.1. GWAS Data and Meta-Analysis

GWAS summary statistics of MDD and insomnia were downloaded from Psychiatric Genomics Consortium (PGC) (Psychiatric Genomics Consortium. Available online: http://www.med.unc.edu/pgc (accessed on 10 December 2020)) and Sleep Disorders Knowledge Portal (SDKP Datasets. Available online: http://kp4cd.org/datasets/sleep (accessed on 10 December 2020)), respectively. The original GWAS studies can be referred to Wray et al. [16] and Lane et al. [21]. Meta-analysis of MDD and insomnia was performed with a sample size-based analytical strategy model using METAL [26]. Specifically, METAL combines *p*-values across studies considering study-specific weights (the sample size) and direction of effect. SNP ID, weight, alleles, frequency, effect size, standard error, and *p*-value were provided from both GWAS summary statistics for METAL to execute.

### 2.2. Identification of Candidate SNPs, Gene Mapping and Functional Annotation

FUMA [27] (v1.3.6) was used to identify candidate SNPs. Linkage disequilibrium (LD) blocks from 1000 Genomes Project Phase 3 [28] EUR population were used as a reference panel to compute r2 and MAF. Candidate SNPs were mapped to genes using positional and eQTL mapping approaches separately. Gene window for positional mapping was set at default maximum distance of 10 kb on both sides and was based on ANNOVAR [29] annotation. Cis-eQTL mapping mapped SNPs to genes up to 1 Mb, and two sets of tissue types were used: (1) whole body tissues in GTEx v8 [30] (54 tissue types, including brain regions), and (2) 13 brain-only regions. Only eQTLs with FDR ≤0.05 were considered statistically significant. Biotypes of mapped genes were annotated by Ensembl BioMart (Ensembl build v92). Functional enrichment analyses were performed using hypergeometric tests. Pathway and functional gene set information was obtained from MSigDB v7.0 [31].

### 2.3. MAGMA Gene-Based Tests

The gene-based analysis was performed using MAGMA [32] v1.08 with SNP-wise mean model as part of the FUMA pipeline. Gene annotation window of 10 kb upstream and 10 kb downstream was used. SNPs were mapped to 19,383 genes obtained from Ensembl build v92 GRCh37. Tissue expression (gene-property) analysis was performed to test the genetic associations of highly expressed genes in a specific tissue based on GTEx v8 [30] RNA-Seq data.

### 2.4. Cell Type Specificity Analysis

MAGMA gene-property analyses were performed to test the relationship between cell type-specific gene expression profiles and phenotype–gene associations. Mouse cerebellar single-cell RNA-Seq data were obtained from DropViz [33]. Primary cell types and their sub-clusters were used for analysis. Human adult brain single-cell data were obtained from GEO Accession GSE67835 [34].

### 2.5. Identification of Druggable Targets

We selected 719 union genes from two mapping strategies and obtained their gene network using the Search Tool for Retrieval of Interacting Genes (STRING v11.5) [35]. Genes were ranked by connectivity degree using the Cytoscape v3.8.2 [36] plugin cytoHubba [37]. To identify druggable targets from these genes, we characterized the drug–gene interactions in the Drug—Gene Interaction Database (DGIdb v4.2.0) [38]. Approved drugs were used as a preset filter. The known targets for MDD and insomnia were acquired using Open Targets Platform v21.06 [39] and then used as input to find their interacted drugs using DGIdb.

## 3. Results

### 3.1. Shared Genetic Variants

**Figure 1:**
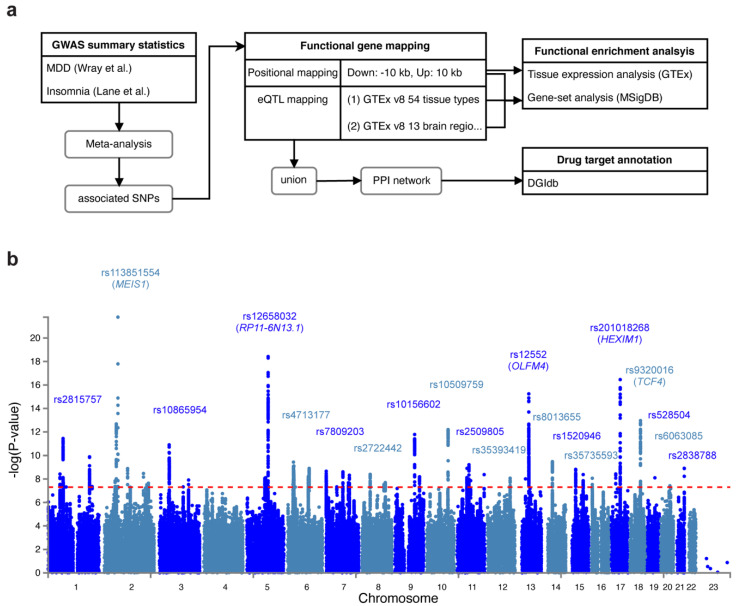
Study flow chart and Manhattan plot for the meta-analysis of MDD and insomnia. (**a**) Flow chart that depicts the workflow of our study. (**b**) Manhattan plot that shows the associated SNPs. The red dashed line indicates the genome-wide significance threshold at *p* = 5 × 10^−8^.

We conducted a genome-wide association meta-analysis of MDD and insomnia based on two previous GWAS studies: (1) Wray et al. [16] identified 44 risk variants in 135,458 major depression cases versus 344,901 controls from seven cohorts. (2) Lane et al. [21] identified 57 loci for self-reported insomnia symptoms in 345,022 cases and 108,357 controls from the UK Biobank. Figure 1a shows the workflow of our analyses. In the meta-analysis, we identified 62 lead variants (*p* < 5 × 10^−8^) at 54 risk loci, among 7062 candidate associated SNPs (*p* < 0.05). The signal (measured by the number of SNPs) was stronger in meta-analysis than in MDD or insomnia study alone (Figure 1b, Supplementary Figure S1, Supplementary Tables S1 and S2). The most significant associated variant was rs113831554 (*p* = 1.64 × 10^−22^), which lies in the intronic region of *MEIS1*, a gene associated with restless legs syndrome (RLS) [40] (Supplementary Figure S2a). This SNP was also reported by Lane et al. [21] with the strongest signal. Furthermore, four other SNPs (rs10156602, rs10865954, rs4577309, and rs12405761) identified by Lane et al. were replicated in our meta-analysis. The second strongest signal we identified was rs12658032 (*p* = 3.77 × 10^−19^), located in the intron of lincRNA *RP11-6N13.1* (Supplementary Figure S2b). This locus was not reported by Wray et al. [16] or Lane et al. [21] but was shown to be associated with MDD and attention deficit/hyperactivity disorder (ADHD) [41]. The third significant SNP was rs12552 (*p* = 5.69 × 10^−16^) in the 3’UTR of *OLFM4*, which was also reported by Wray et al. [16] (Supplementary Figure S2c). The fourth significant SNP was rs201018268 (*p* = 1.2 × 10^−15^), detected in the exonic region of *HEXIM1* (Supplementary Figure S2d), and the fifth associated SNP, rs9320016 (*p* = 1.9 × 10^−13^), was located in the intronic region of *TCF4* (Supplementary Figure S2e). *TCF4* was also mentioned by Wray et al. Yet, the variants they identified in such gene was rs12958048 [16]. Notably, rs201018268, along with other variants: rs558237097, rs575346808, rs8013655, rs1520946, rs529656112, rs75606464, rs35735593, rs71573104, rs6765491, rs17043773, rs62519760, rs12125521, rs12537732, rs12607631, and rs360241, were not previously reported for being associated with any depressive or insomnia traits, suggesting that such variants were new variants shared by MDD and insomnia.

### 3.2. Tissue Expression and Cell Type Specificity

**Figure 2:**
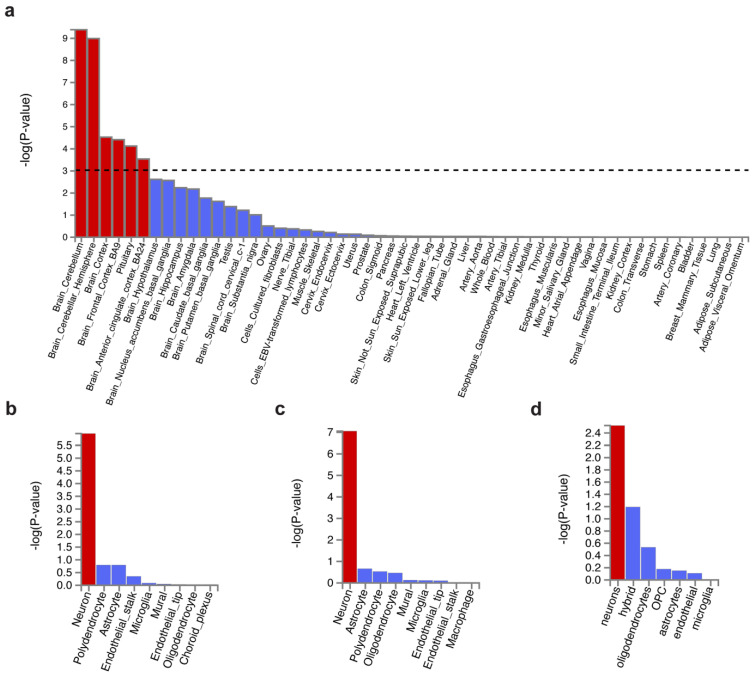
Tissue expression and cell type specificity enrichment of genes associated with shared genetic variants. (**a**) Tissue expression enrichment in GTEx 54 tissue types. The dashed line indicates the significance threshold at *p* = 0.001. (**b**–**d**) Cell type specificity in (**b**) cerebellum, (**c**) frontal cortex BA9, and (**d**) BA24. Bars in red represent significant enrichment.

Tissue enrichment analysis was performed using MAGMA to investigate the tissue expression of variant-associated genes. We found those genes were majorly expressed in the brain but not in the peripheral tissues (Figure 2a). The top enriched brain region was the cerebellum, regardless of hemisphere, followed by the cortex, frontal cortex (FC) BA9, pituitary, and anterior cingulate cortex (ACC) BA24 (Supplementary Table S3). Our results coincide with Wray et al. [16] (MDD enrichment in FC BA9, cortex, and ACC BA24) and Lane et al. [21] (insomnia enrichment in the cerebellum, FC, ACC, and hypothalamus).

Next, we focused on the cell type specificity in the brain regions, including the cerebellum and the frontal cortex (containing Brodmann area BA9 and BA24). It showed that MDD- and insomnia-associated gene expression were enriched in neurons but not glial cells in the cerebellum and the frontal cortex (Figure 2b–d, Supplementary Tables S4–S6). This result was consistent with Wray et al. [16]. Sub-cell type analysis revealed that such cerebellar neurons were both glutamatergic (Slc17a7) and GABAergic (Gad1Gad2) (Supplementary Figure S3a). Interestingly, cortical neurons were predominately enriched in glutamatergic (Slc17a7) (Supplementary Figure S3b).

### 3.3. Gene Mapping and Functional Enrichment

To better understand how these shared variants contribute to the underlying pathophysiology of MDD and insomnia, we have to associate these variants with genes in the genome. Here we adopted two gene mapping strategies: (a) positional mapping based on genomic proximity and (b) eQTL mapping based on linked gene expression across multiple tissues.

**Figure 3:**
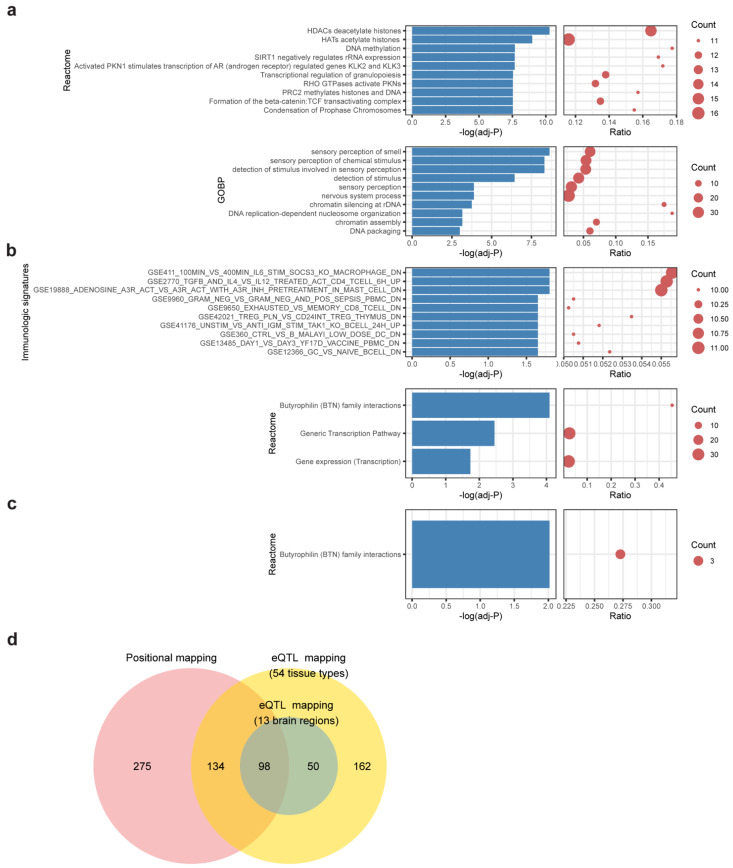
Functional enrichment of shared genes. Bar-dot plots show (**a**) top 10 significant enriched reactome gene sets and top 10 enriched GO biological processes (GOBP) of positional mapping genes; (**b**) enriched reactome gene sets and top 10 enriched immunologic signatures of eQTL mapping genes (54 tissues); (**c**) enriched reactome gene sets of eQTL mapping genes (brain regions). The *p*-value of (**a**–**c**) was FDR-adjusted, and enrichment cutoff was set at adjusted, *p* < 0.05. Count denotes number of genes hit in the respective gene set. Ratio represents the proportion of hit genes to the genes in gene set. (**d**) A Venn diagram showing the relationships of mapped genes under different mapping strategies.

In positional mapping, 507 genes were found proximal to MDD-insomnia shared SNPs (Supplementary Table S7). The largest proportion of the SNPs fell in the intergenic region, followed by genes in the intronic region and then the intronic region of ncRNA (Supplementary Figure S4). The top significant enriched reactome gene sets were HDACs deacetylate histones, HATs acetylate histones, and DNA methylation. The most significant enriched GO biological pathways were sensory perception of smell and sensory perception of chemical stimulus (Figure 3a, Supplementary Table S10).

The eQTL mapping identifies variants associated with gene expression across multiple tissues. The following two contexts were considered in the eQTL mapping: (a) eQTLs found in the whole body tissues (54 tissues in GTEx), and (b) eQTLs found in the brain regions (prefix with Brain in Supplementary Table S3). For (a) whole body tissues, 444 genes were mapped (Supplementary Table S8). These genes are enriched in Butyrophilin (BTN) family interactions and several immunologic signatures, including macrophage transcriptional response to Interleukin-6, CD4+ T cell pathway, and HMC-1 cell activation (Figure 3b, Supplementary Table S11). For (b) brain-only regions, 148 genes were mapped (Supplementary Table S9). Again, the gene set “Butyrophilin family interactions” was enriched (Figure 3c, Supplementary Table S12). Three BTN genes (*BTN3A2*, *BTN2A2*, *BTN3A3*) show strong associations in both mapping contexts.

Those BTN genes also participated in other functions we identified in the enrichment analysis: biological process (*BTN2A2*: regulation of hemopoiesis; *BTN3A2*, *BTN2A2*, *BTN3A1*, *BTN3A3*, and *BTN1A1*: T cell receptor signaling pathway), chemical and genetic perturbation (*BTN3A2*: BROWN_HCMV_INFECTION_48HR_DN; *BTN2A2*: ENK_UV_RESPONSE_EPIDERMIS_UP), and immunologic signatures (*BTN3A2* and *BTN3A1*: GSE1740_UNSTIM_VS_IFNA_STIMULATED_MCSF_DERIVED_MARCOPHAGE_DN; *BTN3A3*: GSE22196_HEALTHY_VS_OBESE_MOUSE_SKIN_GAMMADELTA_CELL_UP; *BTN3A2*, *BTN2A2*, *BTN3A1*, and *BTN3A3*: GSE42021_TCONV_PLN_VS_CD24HI_TCONC_THYMUS_UP; *BTN3A3*: GSE36826_NORMAL_VS_STAPH_AUREUS_INF_IL1R_KO_SKIN_UP).

Some genes from variants we identified and were not previously reported to be associated with MDD or insomnia also participate in immune-related, (epi)genetic, or nervous system function. For example, *HEXIM1* (rs201018268) was involved in GSE41176_UNSTIM_VS_ANTI_IGM_STIM_TAK1_KO_BCELL_24H_UP. *RBFOX1* (rs35735593) was within the gene set of ACEVEDO_LIVER_CANCER_WITH_H3K27ME3_DN. *FOXP2* (rs71573104) was enriched in nervous system process and DNA binding transcription factor activity. The gene *snoU13* (rs12537732) fell in Reactome gene sets generic transcription pathway and gene expression transcription.

Overall, a total of 719 shared genes were identified with at least one of the mapping contexts. Over half (367/719, 51%) of them are protein-coding genes. The remaining half (352/719, 49%) consists of 144 pseudogenes, 66 antisense, 67 lincRNAs, 21 miRNAs, and 54 other biotypes. Of note, shared genes via positional mapping and eQTL mapping are highly overlapped (232 genes in common) (*p* < 5 × 10^−16^, Fisher’s exact test) (Figure 3d), suggesting that a large proportion of shared variants have the potential to influence the expression of proximal genes.

### 3.4. Druggable Targets Identified by Network Approach

**Figure 4:**
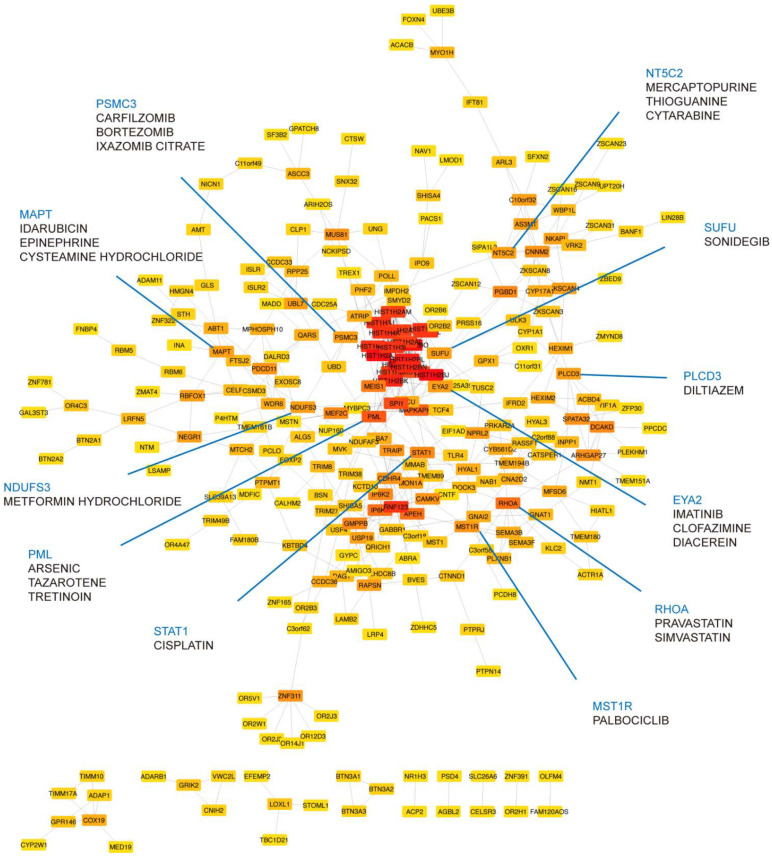
Druggable targets prioritized in the STRING network. A total of 272 shared genes are ordered by their connectivity degree in the network. The color of nodes from red to yellow represents the degree from high to low. Druggable targets in blue are annotated with their potential drugs (drug listed under the target gene name).

Genes associated with MDD and insomnia may provide a list of candidates for finding druggable targets. To this end, we prioritized the identified shared genes according to their protein connectivity in the human protein–protein interaction (PPI) network. A subnetwork consisting of 358 nodes was created using STRING. We then ranked these nodes by their connectivity degree in the network. Among them, 272 genes with connection to others were searched for their potential drugs based on known drug–gene interactions using DGIdb (Supplementary Table S13). Genes with query scores over 6 and their top three interacted drugs were highlighted in Figure 4. Notably, PLCD3 was mapped by one of the novel variants we identified, rs575346808. SUFU was mapped by the lead associated SNP rs12767131 (Supplementary Table S2). Furthermore, RHOA was in the gene set of synapse organization in our GO enrichment result. MST1R was related to pathways involved in H3K4me3 and H3K27me3. STAT1, NT5C2, PML, and EYA2 were enriched in immune-related pathways (Supplementary Tables S10–S12).

To compare the drugs we found with known targets and drugs for both MDD and insomnia, we searched for known targets and drugs for MDD and insomnia in the Open Target database [39] and DGIdb [38]. Among the 1965 unique drugs for MDD and 1535 drugs for insomnia, 1475 drugs are in common, suggesting agents relieve shared symptoms in MDD and insomnia (Supplementary Table S14). Among them, 122 drugs approved for both MDD and insomnia are also reported in our proposed drug list (Table 1), showing the potential of our shared gene strategy and network approach in the application of drug discovery and repurposing.

## 4. Discussion

In this study, we conducted a meta-analysis from GWAS summary statistics of MDD and insomnia and identified common variants underlying the pathophysiology of these two psychiatric conditions. The variants were mapped to genes, and functional enrichment analysis was performed to suggest their functions. We also provide a list of druggable targets and potential drugs for future validation, which may benefit future drug development against the comorbidity of depression and insomnia symptoms.

Our meta-analysis was able to capture the significant signal from both MDD and insomnia summary statistics. We identified 62 variants linked to shared genetics of MDD and insomnia symptoms, with signals near *MEIS1*, *RP11-6N13.1*, *OLFM4*, *HEXIM1*, and *TCF4* being the strongest. Some loci have been reported in previous studies with similar findings. First, a study has reported that *MEIS1* shows a pleiotropy effect on RLS and insomnia [19]. A study using bioinformatics and transgenic mice approaches indicated the regulatory role of MEIS1 in neuropeptide substance *p* expression in the amygdala, suggesting a mechanism underlying anxiety and depression [42]. Second, the *RP11-6N13.1* locus has been implicated in early sleep timing [43] and how it contributes to insomnia remains to be determined. In addition to our finding of rs126580832, rs40465 near *RP11-6N13.1* has been reported to be associated with broad depression [15]. Third, evidence has indicated loci in *OLFM4* are related to major depression [13,44] and insomnia [22]. Fourth, we found a variant rs201018268 located in the exonic region of *HEXIM1*. *HEXIM1* is a transcription regulator suggested for its role in cancer [45,46] and linked to insomnia [21], but its association with MDD still lacks. It is worth further study to investigate whether variation in the exonic region of *HEXIM1* affects MDD phenotype. Fifth, our gene-based analysis showed that *TCF4* was the only gene that reached genome-wide significance in MDD and insomnia. GWAS studies have identified SNPs in *TCF4* susceptible to schizophrenia [47] and corneal endothelial dystrophy [48]. Furthermore, a rare mutation in *TCF4* leads to Pitt–Hopkins syndrome, a rare neurodevelopmental disorder [49,50]. A recent study shows the contribution of *TCF4* in mutual influences between MDD and insomnia [51], which aligns with our result. Although the above loci have been discovered for their associated trait, our study links these risk loci to both MDD and insomnia, suggesting a possible pleiotropy effect on their comorbid phenotypes.

Brain regions and neural network alterations in MDD patients have been found with neuroimaging studies. Specifically, abnormalities in the prefrontal cortex, ACC, amygdala, hippocampus, thalamus, and basal ganglia have been indicated in MDD [52,53,54,55,56]. Moreover, alternations in the inferior frontal gyrus/anterior insula, orbitofrontal cortex, and suprachiasmatic nuclei were found in patients with MDD and co-occurring insomnia [57,58,59]. In line with the evidence above, our results show that genes associated with shared variants are highly expressed in the cortex, FC, ACC, and, surprisingly, the cerebellum. The cerebellum has been recognized to be involved in cognitive and affective functions in addition to functions in motor coordination [60]. Recently, abnormality in cerebellum structure and functions has been reported in depression [61,62]. Sleep disorders, including insomnia, have also been linked to cerebellar malfunction [63,64]. However, none of the research focuses on cerebellum changes in MDD with insomnia, to our knowledge. Therefore, it could be a novel direction to investigate cerebellum in coexisting MDD and insomnia. We also found enriched gene expression of MDD and insomnia in the pituitary, a neuroendocrine gland in the hypothalamic-pituitary-adrenal (HPA) axis. The HPA axis mediates stress response, and its hyperactivity has been implicated in the etiology of MDD, stress-related disorders, and insomnia [65,66,67]. In summary, to better understand the comorbidity of MDD and insomnia, further transcriptomic experiments are needed to identify differentially expressed genes and region-specific transcripts in the aforementioned brain regions.

Our results also suggest that glutamatergic and GABAergic neurons in the cerebellum and glutamatergic neurons in the frontal cortex may play a role in the comorbidity of MDD and insomnia. Indeed, cortical glutamate and GABA dysregulation were observed in MDD and primary insomnia [68,69]. Specifically, patients with MDD have decreased glutamatergic metabolites in the medial frontal cortex [70], whereas subjects with primary insomnia or mood disorder had lower GABA levels in the occipital cortex and ACC [71]. GABA is an inhibitory neurotransmitter, and activation of GABA receptors has been targeted for sleep-promoting agents [72]. Current treatment for depression also showed effects on counteracting GABAergic deficit, increasing hippocampal neurogenesis and maturation [73]. One possible explanation of our excitatory glutamate enrichment results in the frontal cortex could be that the associated gene expression in the glutamatergic neuron down-regulates the glutamate synthesis or release in the frontal cortex. However, its effect on the reduction of GABA transmission and their interplay remains to be explored. On the other hand, although previous studies have suggested a role of glutamate and GABA transmission in MDD and insomnia, none of the studies focus on such neurotransmitter levels in the cerebellum. Our result highlights genetic variation linked to cerebellar excitatory and inhibitory neurotransmission in MDD and insomnia, providing brain region and cell type-specific targets for future research to treat such disorders.

Our functional enrichment analysis reveals that genes linked to MDD and insomnia are involved in several biological pathways. Reactome enrichment of HDACs deacetylate histones, HATs acetylate histones, and DNA methylation together suggests epigenetic effects on these disorders. These results imply that both genetic and epigenetic factors contribute to complex psychiatric disorders. Stressful life experiences were correlated with dysregulation of HDAC2 and HDCA5 levels [74,75], and preclinical studies have suggested HDAC inhibitors as a potential therapeutic agent for MDD [76,77]. Studies also suggested the roles of HDACs in sleep deprivation and melatonin receptors, which are closely related to insomnia [78,79]. Our results also show pathway enrichment in sensory perception of the chemical stimulus, such as smell. This is consistent with evidence showing the correlation between smell and taste alterations in older MDD patients [80]. However, no study so far focuses on the roles of smell perception in insomnia.

One enriched gene set “Butyrophilin family interactions” in our analysis was also significant in a study for major depression using a gene co-expression network-based approach [44]. Butyrophilins (BTNs) are regulators of the immune response. They have both stimulatory and inhibitory effects on immune cells. Specifically, BTN1A1 and BTN2A2 inhibited the proliferation of CD4+ and CD8+ T-cells [81]. They also reduce the expression of various cytokines including IL-2 and IFN-γ [82]. BTN3A1 inhibited T-cell proliferation and cytokine production, leading to caspase-8 silencing [83]. BTN3A also inhibited apoptosis for the increased survival of monocytes and dendritic cells, enhancing the synthesis of IL-1, IL-8, and IL-12 [84]. Together with our enrichment in several immunologic signatures, our result indicated that MDD with insomnia is associated with a dysfunctional immune system. Accumulating evidence has suggested that MDD is linked to elevated proinflammatory cytokines such as IL-1, IL-6, tumor necrosis factor (TNF)-α, and chemokines [85,86,87,88]. Therefore, we hypothesized that IL-1 could be a link for the mechanism of MDD through BTN3A. Studies also indicated that the phase shift of IL-6 and TNF secretion is associated with chronic insomnia [89]. These findings revealed that anti-inflammatory drugs might become promising medications for treating MDD with insomnia. Our result supports the previous finding of immune dysregulation in MDD with insomnia and provides a new direction in studying epigenetic or sensory perception-related pathways in this disorder.

Recent pharmacological treatments for MDD with insomnia include antidepressants with sleep-promoting properties, such as Mirtazapine [90]. The action of such antidepressants has long been based on the “monoamine hypothesis”, most of which acts on 5-HT (serotonin), norepinephrine, or histamine receptors [91,92]. However, antidepressant medications can be non-effective and hardly improve the subjective rating of sleep quality [93]. Although alternative agents can be prescribed for resistant insomnia in depression, for example, benzodiazepine drugs or melatonin, drug dependence and their modest effects have been a concern, respectively [94,95,96]. This study identified potential druggable targets for MDD with insomnia and their existing drugs for drug repurposing. However, some limitations existed. MDD and insomnia are polygenic disorders; hence, it is difficult to quantify the single gene contribution, and drug design for multi-targets has been challenging. Although we prioritized the targets based on their interaction with other proteins, it does not equate to a higher contribution to the disorders’ pathophysiology. Further studies and technologies are demanded to conquer the above problems to improve drug development for MDD with insomnia.

## 5. Conclusions

In conclusion, we identified risk loci that link to individuals’ susceptibility to developing MDD with insomnia. Our analyses further revealed tissue and cell type-specific gene expression associated with these two disorders. Functional enrichment analysis suggested pathways in epigenetic, sensory perception, and immune functions in MDD with insomnia. Finally, we provided a list of druggable targets and potential drugs for future medication in treating comorbid MDD and insomnia conditions.

## Figures and Tables

**Table 1:** List of proposed drugs with use approvals for both MDD and insomnia.

Name of Drug		
Abiraterone Acitretin Alcohol Alendronic Acid Alprostadil Aminohippuric Acid Amodiaquine Apomorphine Atenolol Baclofen Bepridil Hydrochloride Bortezomib Butabarbital Butalbital Butethal Capecitabine Carbamazepine Carbidopa Carboplatin Carfilzomib Cefaclor Cholecalciferol Cisplatin Cyclosporine Cysteamine Hydrochloride Cytarabine Dacarbazine Dantrolene Dantrolene Sodium Daunorubicin Daunorubicin Hydrochloride Deferasirox Dexamethasone Dexketoprofen Diacerein Didanosine Dihydroergotamine Dihydroergotamine Mesylate Diltiazem Docetaxel Doxorubicin	Doxorubicin Hydrochloride Enzalutamide Epinephrine Epinephrine Bitartrate Ethopropazine Hydrochloride Felodipine Gabapentin Gabapentin Enacarbil Gefitinib Gemcitabine Gentian Violet Granisetron Hexachlorophene Hydroxyzine Pamoate Idarubicin Imatinib Inamrinone Infliximab Itraconazole Lansoprazole Menadione Mephobarbital Mercaptopurine Mesalamine Mesna Metformin Metformin Hydrochloride Metharbital Methotrexate Methylene Blue Mitoxantrone Hydrochloride Mycophenolate Mofetil Mycophenolic Acid Nelfinavir Niclosamide Nifedipine Nifuroxazide Nitazoxanide Norepinephrine Olanzapine Omeprazole	Oxitriptan Oxytetracycline Oxytetracycline - Hydrochloride Palbociclib Pantoprazole Phenazopyridine - Hydrochloride Phenobarbital Phenytoin Pravastatin Pregabalin Primidone Progesterone Promethazine Pyrantel Pamoate Rabeprazole Raloxifene Raloxifene -Hydrochloride Ribavirin Risperidone Ritonavir Safinamide Saquinavir Simvastatin Sodium Oxybate Sonidegib Spironolactone Sulfasalazine Tacrolimus Talbutal Tazarotene Thioguanine Thiopental Topiramate Trastuzumab Tretinoin Triclabendazole Trimetrexate Verapamil Vigabatrin Warfarin

## Data Availability

The data presented in this study are available online: MDD GWAS Summary Statistics, https://www.med.unc.edu/pgc/download-results/mdd/ accessed on 10 December 2020; Insomnia GWAS Summary Statistics, http://www.kp4cd.org/dataset_downloads/sleep accessed on 10 December 2020; METAL, https://genome.sph.umich.edu/wiki/METAL; FUMA GWAS, https://fuma.ctglab.nl/ accessed on 10 December 2020.

## Supplementary Materials

The following are available online at https://www.mdpi.com/article/10.3390/genes12101506/s1, Figure S1: Manhattan plot and Q-Q plot of MDD and insomnia, Figure S2: Regional association plots of the top 5 variants, Figure S3: Second level of cell type specificity analysis, Figure S4: Functional consequences of SNPs on genes in positional mapping, Supplementary Table S1: Summary of the datasets used in this study; Supplementary Table S2: Lead SNP results from meta-analysis; Supplementary Table S3: Results from tissue expression analysis; Supplementary Table S4: Results from cell type specificity analysis in the mouse cerebellum; Supplementary Table S5: Results from cell type specificity analysis in the mouse frontal cortex; Supplementary Table S6: Results from cell type specificity analysis in the human cortex; Supplementary Table S7: Results from positional mapping; Supplementary Table S8: Results from eQTL mapping (54 tissue types); Supplementary Table S9: Results from eQTL mapping (13 brain regions); Supplementary Table S10: Gene set analysis results from positional mapping; Supplementary Table S11: Gene set analysis results from eQTL mapping (54 tissue types); Supplementary Table S12: Gene set analysis results from eQTL mapping (13 brain regions); Supplementary Table S13: Protein connectivity ranking and the drug-gene interaction results; Supplementary Table S14: Approved shared drugs for MDD and insomnia.
