# Supplementary material for: GWAS Meta-Analysis Reveals Shared Genes and Biological Pathways between Major Depressive Disorder and Insomnia
Source: Genes (Basel). 2021 Sep 26;12(10):1506. doi: 10.3390/genes12101506 (PMC8536096; doi:10.3390/genes12101506)
Supplement: Supplementary file 1 [file genes-12-01506-s001.zip › Figure S1-S4_R1.pdf]

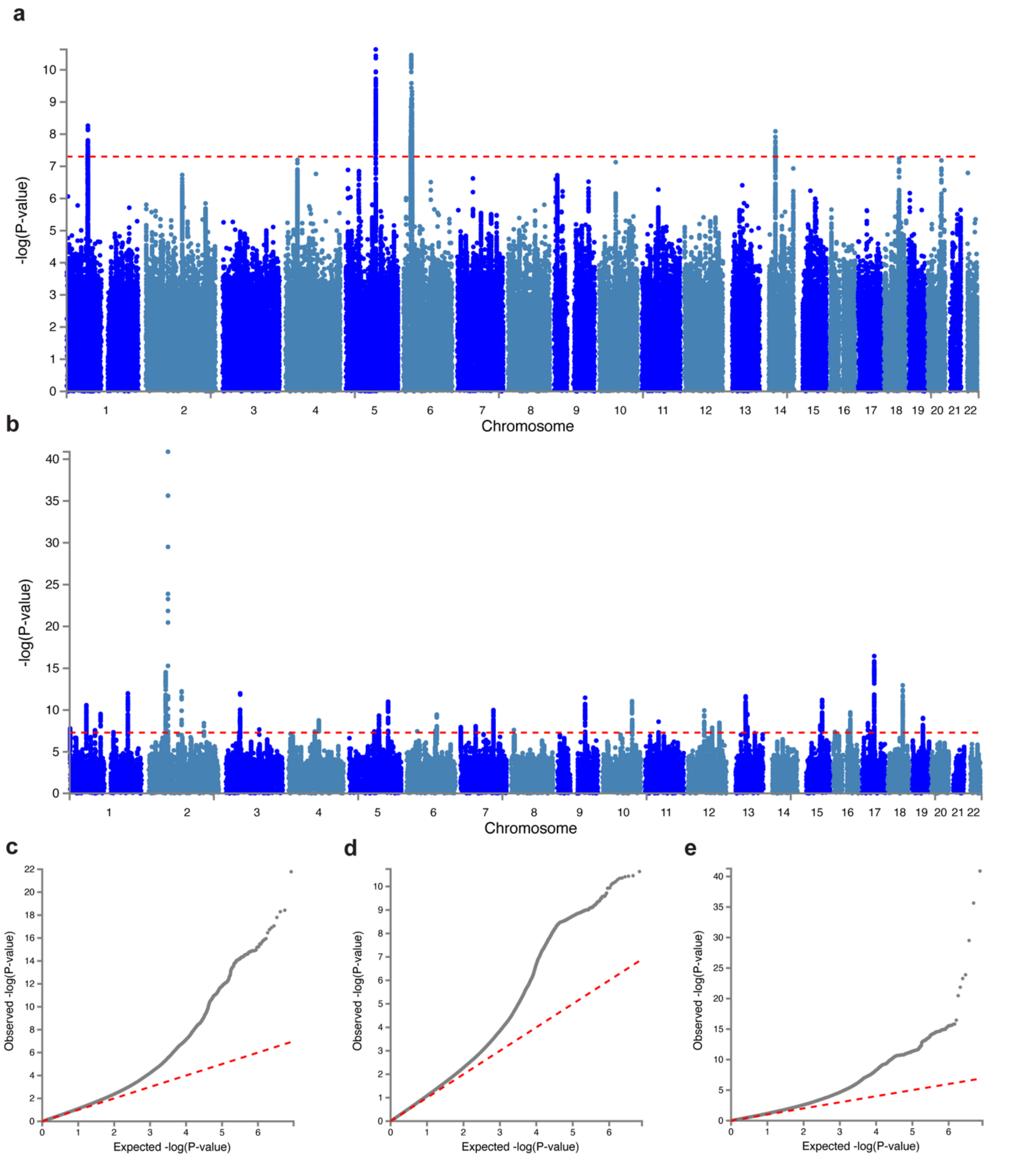

Figure S1. Manhattan plot and Q-Q plot of MDD and insomnia. Manhattan plot shows the associated SNPs of **a.** MDD and **b.** insomnia from the GWAS summary statistics from Wray et al. and Lane et al., respectively. The red dashed line indicates the genome-wide significance threshold at P = 5e-8. **c.** Q-Q plot of the meta-analysis of MDD and insomnia. Q-Q plot of MDD is shown in **d**, and that of insomnia is shown in **e**.

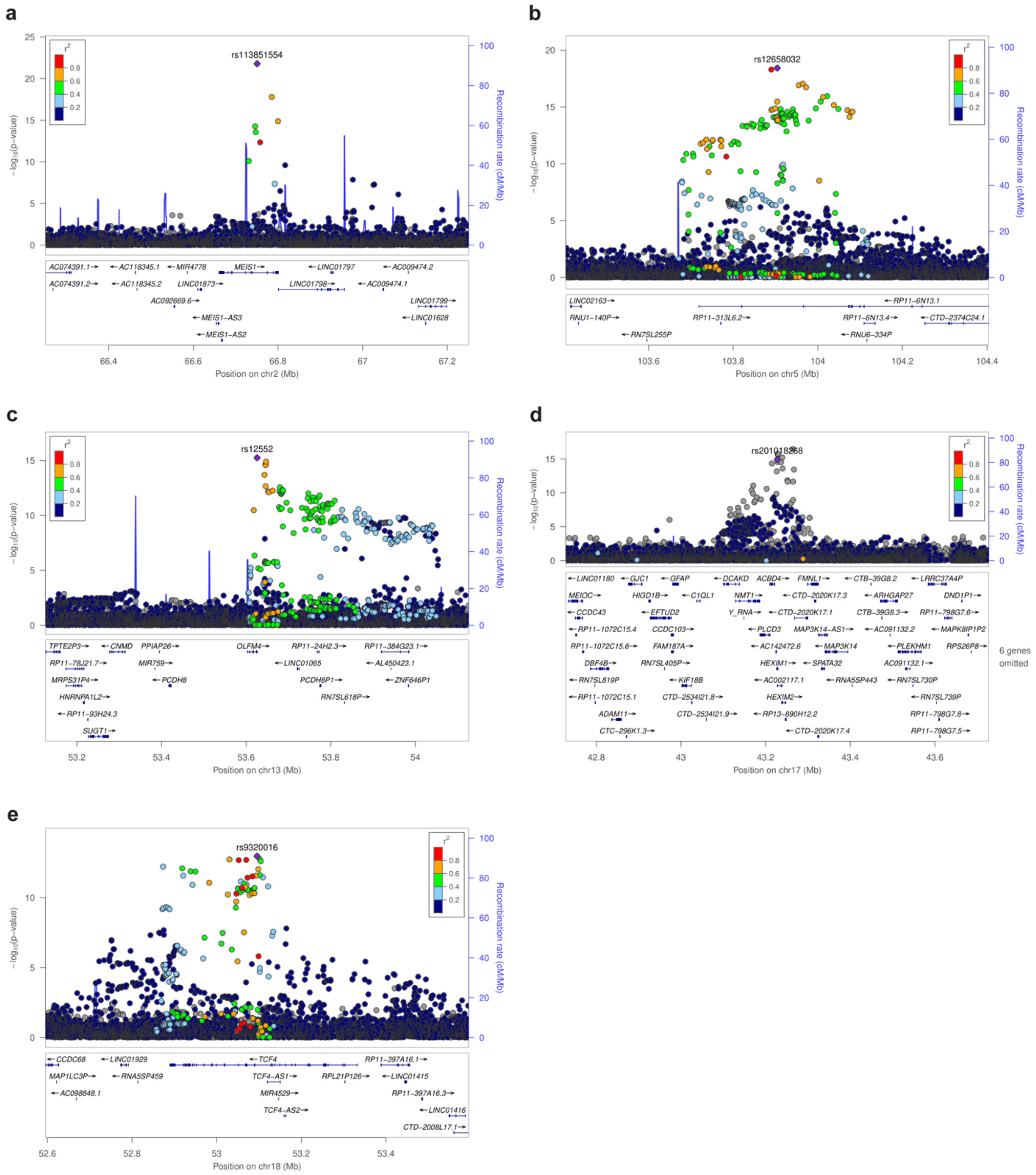

Figure S2. Regional association plots of the top 5 variants. Regional association of **a.** rs113851554, **b.** rs12658032, **c.** rs12552, **d.** rs201018268 and **e.** rs9320016 are shown.

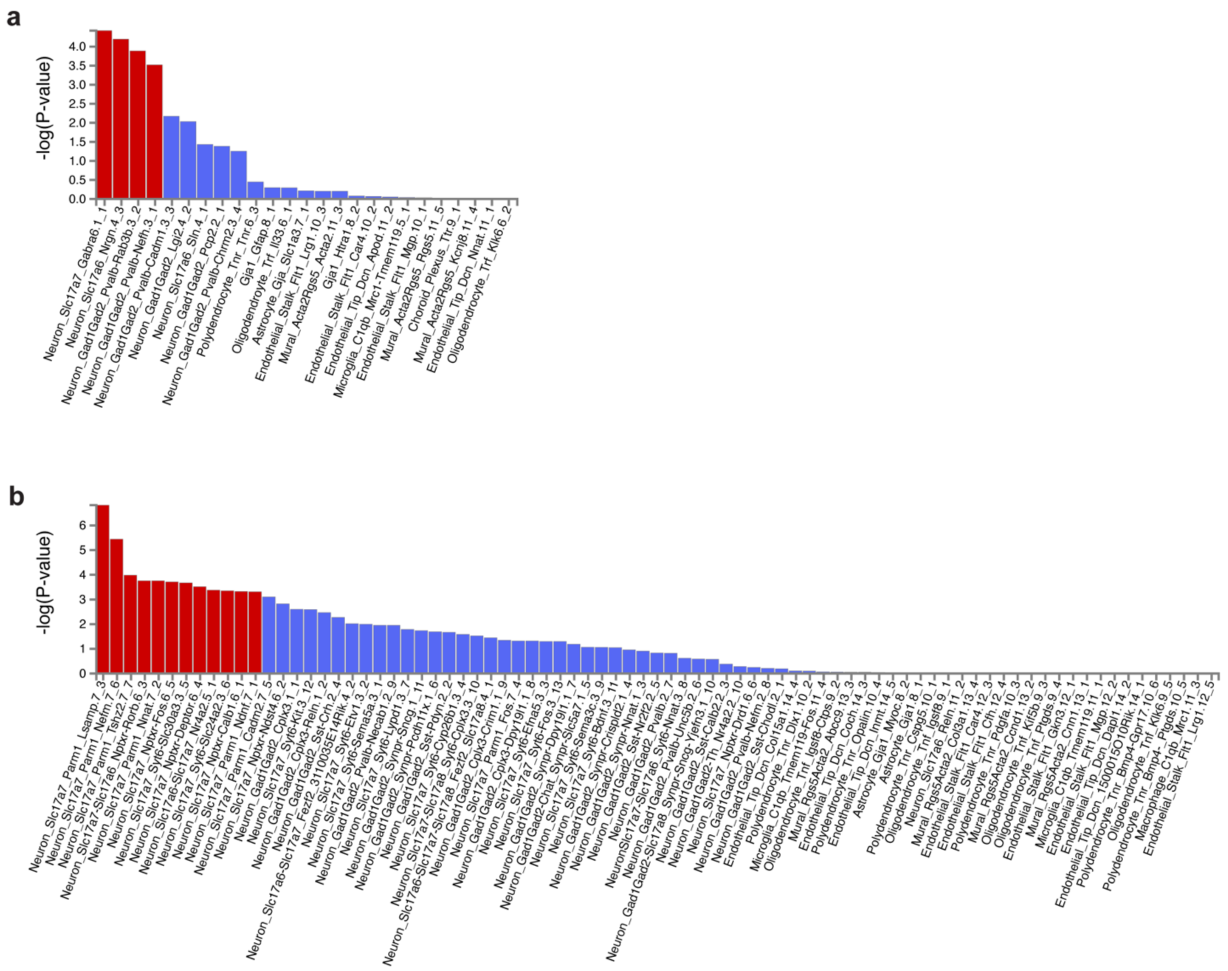

Figure S3. Second level of cell type specificity analysis. a. Result with mouse cerebellum. **b.** Result with mouse frontal cortex. Bars in red represent significant enrichment.

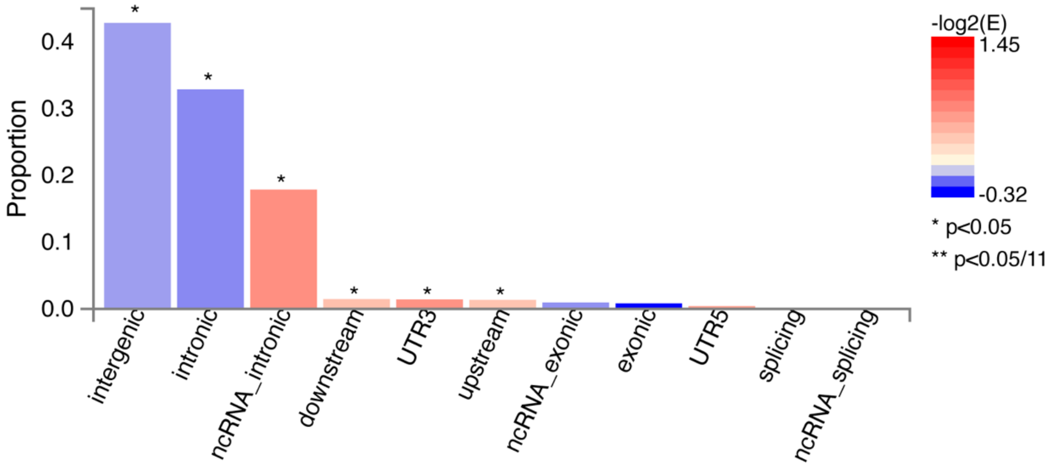

Figure S4. Functional consequences of SNPs on genes in positional mapping. The horizontal axis indicates different functional consequences. The vertical axis shows the proportion. Colors code enrichment level and direction. Stars represent significant at *P* < 0.05.
